# Genic SNP markers and legume synteny reveal candidate genes underlying QTL for *Macrophomina phaseolina *resistance and maturity in cowpea [*Vigna unguiculata *(L) Walp.]

**DOI:** 10.1186/1471-2164-12-8

**Published:** 2011-01-05

**Authors:** Wellington Muchero, Jeffrey D Ehlers, Timothy J Close, Philip A Roberts

**Affiliations:** 1 Nematology Dept., University of California-Riverside, 3401 Watkins Drive, Riverside, CA 92521, USA; 2 Botany and Plant Sciences Dept., University of California-Riverside, 3401 Watkins Drive, Riverside, CA 92521, USA; 3 Current Address: Plant Systems Biology Group, Bioscience Division, Oak Ridge National Laboratory, 1 Bethel Valley Road, Oak Ridge, TN 37830, USA

## Abstract

**Background:**

*Macrophomina phaseolina *is an emerging and devastating fungal pathogen that causes significant losses in crop production under high temperatures and drought stress. An increasing number of disease incidence reports highlight the wide prevalence of the pathogen around the world and its contribution toward crop yield suppression. In cowpea [*Vigna unguiculata *(L) Walp.], limited sources of low-level host resistance have been identified, the genetic basis of which is unknown. In this study we report on the identification of strong sources of host resistance to *M. phaseolina *and the genetic mapping of putative resistance loci on a cowpea genetic map comprised of gene-derived single nucleotide polymorphisms (SNPs) and amplified fragment length polymorphisms (AFLPs).

**Results:**

Nine quantitative trait loci (QTLs), accounting for between 6.1 and 40.0% of the phenotypic variance (R^2^), were identified using plant mortality data taken over three years in field experiments and disease severity scores taken from two greenhouse experiments. Based on annotated genic SNPs as well as synteny with soybean (*Glycine max*) and *Medicago truncatula*, candidate resistance genes were found within mapped QTL intervals. QTL *Mac-2 *explained the largest percent R^2 ^and was identified in three field and one greenhouse experiments where the QTL peak co-located with a SNP marker derived from a pectin esterase inhibitor encoding gene. Maturity effects on the expression of resistance were indicated by the co-location of *Mac-6 *and *Mac-7 *QTLs with maturity-related senescence QTLs *Mat-2 *and *Mat-1*, respectively. Homologs of the *ELF4 *and *FLK *flowering genes were found in corresponding syntenic soybean regions. Only three *Macrophomina *resistance QTLs co-located with delayed drought-induced premature senescence QTLs previously mapped in the same population, suggesting that largely different genetic mechanisms mediate cowpea response to drought stress and *Macrophomina *infection.

**Conclusion:**

Effective sources of host resistance were identified in this study. QTL mapping and synteny analysis identified genomic loci harboring resistance factors and revealed candidate genes with potential for further functional genomics analysis.

## Background

*Macrophomina phaseolina *(Tassi) Goid. is a soil-borne deuteromycete fungal pathogen with a worldwide distribution and a host range that includes more than 500 crop and non-crop plant species [1]. The fungus is a generalist pathogen that attacks stressed plants at all stages of growth causing charcoal rot, seedling damping-off, and ashy stem blight diseases of major and minor crops [1]. Major crops such as soybean, maize, and sorghum as well as some crops of economic importance such as common bean are all known hosts of the pathogen and disease incidence is often associated with high temperatures and drought stress. Recently, there has been a worldwide increase in reports of incidence of the pathogen on diverse crop species [2-8], which could reflect a wider appreciation of the importance of this disease to crop production in drought-prone regions.

In the arid sub-Saharan region of West Africa where cowpea is a crop of major economic importance for resource poor farmers, seedling damping-off and ashy stem blight diseases of cowpea caused by *Macrophomina *result in significant yield losses [9]. Cowpea is a major crop in this and other arid regions due, in part, to its comparatively high capacity to withstand drought stress and poor soil conditions [10]. The sub-Saharan cowpea production region is characterized by high temperatures (average maximum daily > 30°C) and intermittent rainfall with periods of drought which favor disease development [11]. As a result, cowpea yields remain low (0.2-0.5 t/ha) with *Macrophomina *infection under drought stress being a major yield-suppressing factor in this agro-ecological zone.

The synergism between disease severity and drought stress is important in breeding programs which are developing drought tolerant cultivars. In cowpea, as in other crops, significant investments continue to be made in the genetic improvement of crop productivity under limited water conditions [12]. However, relatively little emphasis has been placed on the impact of *Macrophomina *on elite drought-tolerant cultivars in breeding programs. In evaluations of drought-tolerant cowpea genotypes, Ndiaye [11] reported that they were all susceptible to *Macrophomina *infection. With the exception of the stay-green trait in sorghum [13], there are no reports of genetic overlap between drought tolerance and resistance to *Macrophomina*.

Host plant resistance-based management of *Macrophomina *is a potential option for resource-poor farmers. However, in cowpea, only minor sources of resistance have been reported among a few genotypes evaluated in India [14] and Senegal [15], and no studies have identified genomic regions involved in resistance against the pathogen. In other crops, potentially useful minor sources of resistance have been reported in sorghum [16-18], soybean [19,20], and common bean [21,22]. Further, studies in common bean suggested that late-maturing varieties were more resistant to attack by *Macrophomina *than early-maturing varieties [22], and similar observations were made in soybean [19] and sorghum [23].

The purpose of this study was to evaluate the levels of resistance against *Macrophomina *infection in a diverse set of fourteen cowpea genotypes as well as identifying and mapping quantitative trait loci (QTL) associated with resistance to *Macrophomina *infection in a recombinant inbred line (RIL) population, IT93K-503-1 × CB46. We used a genetic map developed by merging an AFLP-based map [24] and an expressed sequence tag (EST)-derived single nucleotide polymorphism (SNP) marker-based map [25] for the same population. A consensus genetic linkage map [25] incorporating SNP markers from the IT93K-503-1 × CB46 map and five other RIL populations was utilized for synteny-based candidate gene identification and definition of QTL locations on the cowpea map. Since the IT93K-503-1 × CB46 population was used previously to map QTL for the delayed drought-induced premature senescence and maturity-related senescence traits [24], the extent of genetic overlap between drought tolerance, maturity, and *Macrophomina *resistance loci in cowpea was also evaluated.

## Results

### Experimental conditions and disease development

Sufficient disease incidence for differentiation of cowpea genotypes was observed in drought-stressed field experiments conducted in each of the three years. The historically high incidence of *Macrophomina*-induced ashy stem blight on cowpea in the plots used for the experiments coupled with high temperatures and lack of significant precipitation in the study area enabled adequate disease development to facilitate phenotyping. Average maximum daily temperatures during the experimental periods were 33, 32, and 33°C for the 2006, 2007, and 2008 seasons, respectively, whereas average minimum temperatures were 17, 16, and 17°C, respectively. For the same periods, total precipitation received was 1.0, 0.5, and 6.1 mm, respectively.

### Phenotyping 14 diverse genotypes

**Figure 1:**
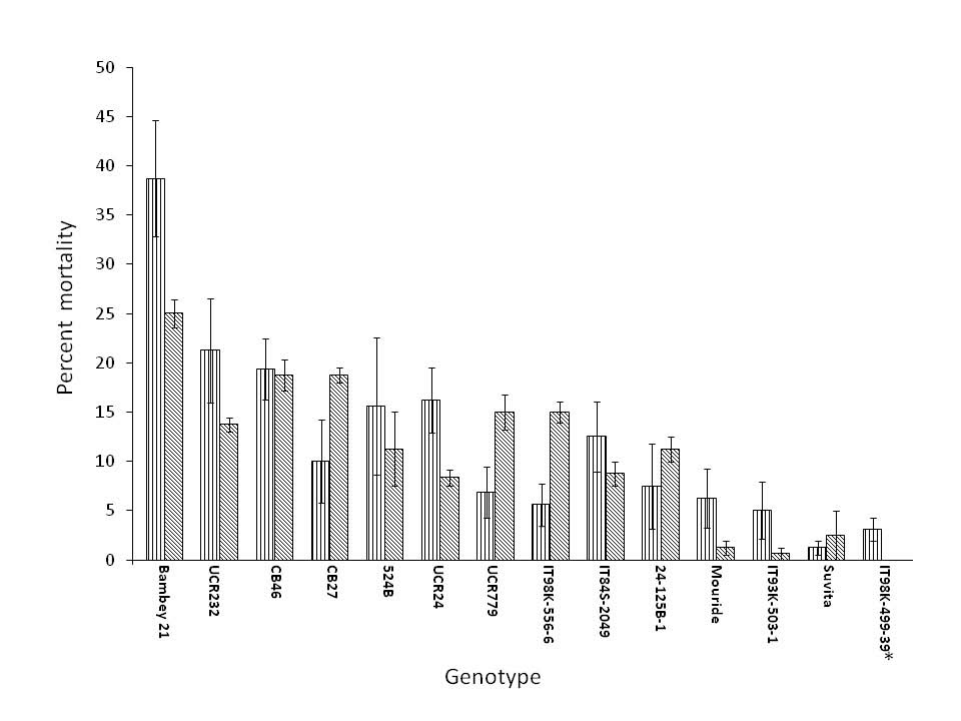
**Percent plant mortality for fourteen diverse cowpea genotypes exposed to *Macrophomina phaseolina *under drought stress in field experiments conducted at the University of California Riverside in 2006 (vertical) and 2007 (slanted)**. See text for complete genotype designations. Bars indicate ± standard error. * Mortality for IT98K-499-39 was zero in the 2007 experiment.

Based on percent plant mortality, there were reproducible differences in genotypic response to drought-enhanced *Macrophomina *infection among the diverse set of fourteen cowpea genotypes over the two experiments conducted in 2006 and 2007 (Figure 1). Genotypes IT98K-499-39, Suvita 2, IT93K-503-1 and Mouride were the most resistant to disease development with mortality less than 10% in both experiments (Figure 1). Bambey 21 was the most susceptible genotype with a mortality of 25-39%, followed by CB46, UCR232 (IT82E-18), 524B, UCR24, and IT84S-2049. Other genotypes exhibited varying levels of intermediate response over both experiments (Figure 1).

### Phenotyping RILs

**Figure 2:**
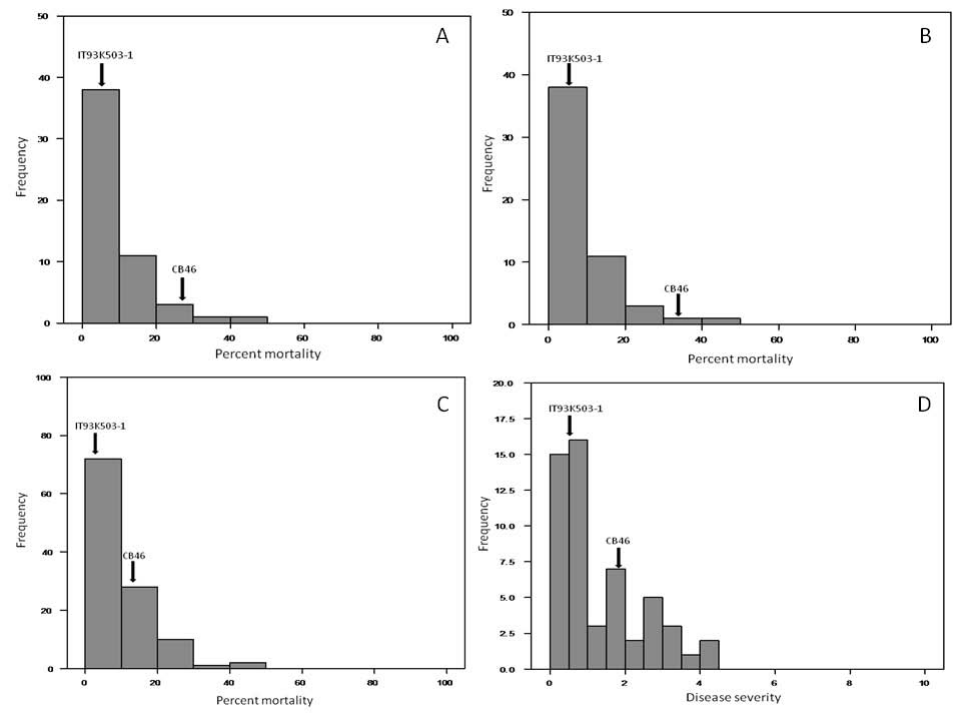
**Frequency distribution for percent plant mortality related to drought-enhanced *Macrophomina phaseolina *infection under field conditions in 2006 (A), 2007 (B), and 2008 (C): and drought-enhanced disease severity ratings under greenhouse conditions averaged over two experiments (D) in a population of recombinant inbred lines developed from a cross between homozygous genotypes IT93K-503-1 and CB46**.

For the IT93K-503-1 × CB46 RIL population, there was significant correlation between all three field experiments for percent plant mortality based on Spearman Rank analysis (r ≥ 0.3643). Further, both greenhouse experiments were correlated (r = 0.6217) for disease severity, however correlations between field and greenhouse experiments were mostly insignificant with the exception of the field experiment conducted in 2007 and greenhouse experiment 1 (Table 1). Percent mortality and disease severity phenotypic data from all five experiments deviated from normality and there was evidence of transgressive segregation for both resistance and susceptibility to *Macrophomina *infection indicated by mean phenotypic scores of some lines lying outside the range of the parents (Figure 2).

**Table 1:** Spearman Rank correlations for percent mortality and disease severity ratings

	2006	2007	2008	Greenhouse 1
2007	0.5508****			
2008	0.3643**	0.3995**		
Greenhouse 1	0.1576 ^ns^	0.3377*	0.1332 ^ns^	
Greenhouse 2	0.1844 ^ns^	0.2485 ^ns^	0.0477 ^ns^	0.6217****

ns = P > 0.05, * = P < 0.05, ** = P < 0.01, *** = P < 0.001, **** P < 0.0001

### QTL mapping, associated SNP markers, and synteny

**Figure 3:**
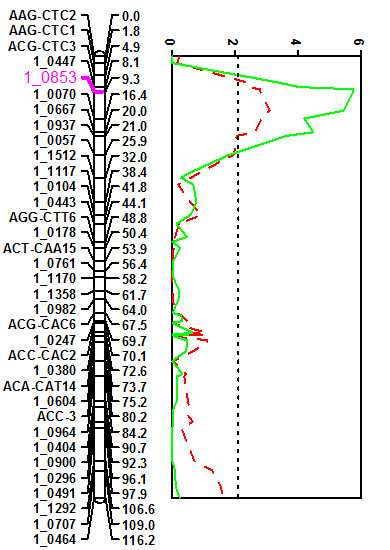
**LOD traces for *Macrophomina phaseolina *resistance QTL *Mac-2 *based on mortality collected in 2006 (broken red line) and 2007 (solid green line)**. Traces are shown for two experiments where the LOD scores exceeded the significance threshold based on the permutation test (Table 2).

Eight QTL regions distributed over four linkage groups of the cowpea consensus genetic map were detected in at least two different experiments based on the criteria described in the materials and methods. Seven of the eight QTL derived their favorable allele from the resistant IT93K-503-1 parental genotype whereas the favorable allele for QTL *Mac-8 *was derived from the susceptible CB46 parental genotype. An additional suggestive QTL, *Mac-4*, did not meet the significance criteria, but was included because peaks were observed at the 63.0 cM and 64.2 cM positions of RIL population linkage group 3 in three separate experiments (Table 2). QTL *Mac-2 *had the highest significance level as well as R^2^. The same interval was mapped in all three field and one greenhouse experiments, meeting the Kruskal-Wallis significance threshold of 0.005 in each case (Table 2). LOD traces of *Mac-2 *QTL are shown in Figure 3 for two experiments based on results of the MQM analysis. The R^2 ^for this QTL ranged between 8.0% and 40% across four experiments. The QTL peak co-located with SNP marker 1_0853 derived from an EST with a pectin esterase inhibitor annotation. This marker mapped to linkage group 3 of the cowpea consensus map. Comparative genome analysis revealed extensive synteny between the cowpea region carrying the 1_0853 marker on linkage group 3 and a segment of *M. truncatula *chromosome 4 [25]. Within the *M. truncatula *region, two copies of pectin esterase inhibitor gene and a pectin esterase virulence target gene exist. Similarly, the corresponding soybean genomic region on chromosome 8 harbors a pectin esterase inhibitor and two copies of a pectin esterase encoding gene. A separate QTL, *Mac-3*, mapped on the same cowpea linkage group and co-located with SNP marker 1_0604 derived from an EST with a pectin acetylesterase gene annotation (Table 2).

**Table 2:** QTL mapping results based on Kruskal-Wallis (KW) and Multiple-QTL Model mapping (MQM) analysis

						KW	MQM			
								
Experiment	QTL	**LG**^**†**^	QTL interval	**Consensus LG and map position (cM)**^**†**^	Marker closest to QTL peak	Significance level	LOD	LOD threshold	***R***^**2**^	Marker annotation
Riverside 2007	*Mac-1*	2	5.2 - 21.8	2 (77.4)	1_0709	0.01	1.77	2.0	14.5	MATE efflux family protein
Greenhouse 1	*Mac-1*	2	6.8 - 21.8	2 (75.2)	1_0551	0.005	2.74	2.0	20.9	Endo-xyloglucan transferase
Greenhouse 2	*Mac-1*	2	5.2 - 21.8	2 (77.4)	1_0709	0.05	1.82	2.0	14.4	MATE efflux family protein
Riverside 2006	*Mac-2*	3	8.1 - 25.9	3 (1.3)	1_0853	0.001	3.11	2.1	26.5	Pectin esterase inhibitor
Riverside 2007	*Mac-2*	3	8.1 - 25.9	3 (1.3)	1_0853	0.0001	5.76	2.1	40.0	Pectin esterase inhibitor
Riverside 2008	*Mac-2*	3	8.1 - 25.9	3 (1.3)	1_0853	0.005	1.73	2.1	8.0	Pectin esterase inhibitor
Greenhouse 1	*Mac-2*	3	8.1 - 25.9	3 (1.3)	1_0853	0.005	1.45	2.1	11.6	Pectin esterase inhibitor
Riverside 2006	*Mac-3*	3	68.3 - 84.2	3 (42.3)	1_0604	0.005	1.24	2.1	10.6	Pectin acetylesterase precursor
Riverside 2007	*Mac-3*	3	58.2 - 84.2	3 (42.3)	1_0604	0.005	1.16	2.1	9.7	Pectin acetylesterase precursor
Riverside 2006	*Mac-4^¶^*	3	109.0 - 116.2	3 (64.2)	1_0464	0.05	1.61	2.1	13.3	Ribosomal protein L7Ae
Riverside 2007	*Mac-4^¶^*	3	109.0 - 116.2	3 (63.0)	1_0201	0.05	1.18	2.1	9.9	Cell growth defect factor 1
Riverside 2008	*Mac-4^¶^*	3	97.9 - 116.2	3 (63.0)	1_0201	0.05	1.21	2.1	6.1	Cell growth defect factor 1
Riverside 2006	*Mac-5*	11	6.1 - 14.4	3 (-)	ACA-CAT13^‡^	0.005	2.26	1.5	18.1	-
Riverside 2007	*Mac-5*	11	6.1 - 14.4	3 (70.8)	1_0079	0.01	1.22	1.5	10.3	CA2+- binding protein 1
Riverside 2008	*Mac-5*	11	9.1 - 13.9	3 (74.0)	1_0496	0.1	1.40	1.5	6.7	MLP-like protein 423
Riverside 2006	*Mac-6*	5	17.2 - 23.2	4 (57.5)	1_0699	0.01	2.27	2.1	18.2	Tropine dehydrogenase
Riverside 2007	*Mac-6*	5	8.9 - 23.2	4 (59.3)	1_0804	0.005	1.99	2.1	16.2	Light harvesting complex PSII
Riverside 2008	*Mac-6*	5	7.4 - 20.5	4 (-)	ACT-CAT8	0.05	1.61	2.1	7.7	-
Riverside 2006	*Mac-7*	5	31.8 - 49.1	4 (41.0)	1_0678	0.01	2.44	2.1	19.4	UDP-glycosyltransferase
Riverside 2007	*Mac-7*	5	27.5 - 49.1	4 (41.0)	1_0678	0.0005	2.44	2.1	19.4	UDP-glycosyltransferase
Riverside 2008	*Mac-7*	5	41.2 - 53.8	4 (34.2)	1_0153	0.0005	2.91	2.1	13.3	lipase class 3 family protein
Riverside 2006	*Mac-8*	6	28.4 - 40.0	5 (-)	AAG-CTC9	0.005	2.20	2.1	18.0	-
Riverside 2007	*Mac-8*	6	27.9 - 43.3	5 (29.7)	1_0030	0.05	1.01	2.1	8.6	60S ribosomal protein L10
Riverside 2008	*Mac-8*	6	29.8 - 41.2	5 (29.7)	AAG-CTC9	0.005	2.64	2.1	12.1	-
Greenhouse 1	*Mac-8*	6	36.1 - 44.5	5 (29.7)	1_0030	0.05	1.23	2.1	9.9	60S ribosomal protein L10
Riverside 2008	*Mac-9*	6	53.5 - 63.2	5 (44.6)	1_0032	0.005	2.12	2.1	12.1	Protein transport protein SEC61
Greenhouse 1	*Mac-9*	6	53.5 - 71.4	5 (40.9)	1_1533	0.05	1.29	2.1	10.4	Integral membrane Yip1 family protein
Greenhouse 2	*Mac-9*	6	53.5 - 70.7	5 (40.9)	1_1533	0.05	1.01	2.1	8.3	Integral membrane Yip1 family protein

^† ^LG = IT93K-503-1 × CB46 RIL population linkage group based on AFLP + SNP map, Consensus LG = corresponding linkage group in the cowpea consensus genetic map, consensus map position represents position of the marker closest to the QTL peak based on mapping in the RIL population

^‡ ^cM position on the consensus map and annotations for AFLP markers were not determined

*^¶ ^*Mac-4 was included as a suggestive QTL since it did not meet the significance thresholds in either the Kruskal-Wallis or MQM analysis

QTL *Mac-1 *was identified in two field and both greenhouse experiments with R^2 ^estimates between 14.4% and 20.9%. This QTL mapped within 2.2 cM in three experiments (Table 2). The most consistent loci for this QTL on cowpea linkage group 2 coincided with a MATE efflux family protein encoding gene and was highly syntenic to homeologous regions on *G. max *chromosomes 10 and 20. The region on soybean chromosome 10 harbored nine copies of the MATE efflux family protein whereas the chromosome 20 region carried three copies of the gene.

**Figure 4:**
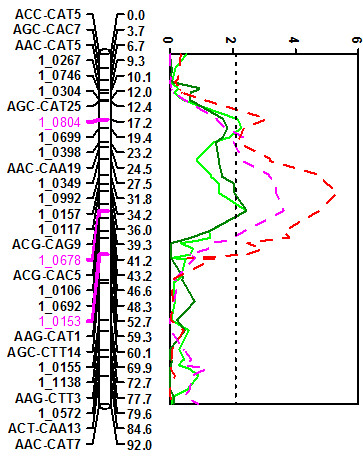
**LOD traces showing the co-location of *Macrophomina *resistance QTL *Mac-6 *and *Mac-7 *(solid green lines) with maturity-related senescence QTL *Mat-1 *and *Mat-2 *(broken red and magenta lines).** Candidate SNP markers are highlighted in magenta color, vertical broken lines illustrate the LOD significance threshold.

QTLs *Mac-6 *and *Mac-7 *co-located with maturity-related senescence QTLs *Mat-2 *(AFLP marker interval AGC-CAC7 - AAC-CAA19) and *Mat-1 *(AFLP marker interval ACG-CAG9 - AGG-CAT1), respectively [24], (Figure 4). In both cases, the *Macrophomina *resistance allele was associated with lateness in maturity.

For *Mac-6*, accounting for between 7.7% and 18.2% of the phenotypic variance for response to *Macrophomina *infection, the SNP marker 1_0804, derived from a homolog of the light harvesting complex PSII (*LHCB4.3*) gene, co-located with the QTL peak in two of the three experiments in which the QTL was identified (Figure 4). The cowpea region harboring *Mac-6 *revealed macrosynteny with sections on soybean chromosomes 11 and 18 where copies of the *LHCB4.3 *gene were found [25]. Within the soybean chromosome 11 region, a homolog of the arginyltransferase (*ATE1*) delayed senescence gene as well as a homolog of the *ELF4 *(Early Flowering 4) gene were found contiguous to the *LHCB4.3 *locus (Table 3) ftp://ftp.jgi-psf.org/pub/JGI_data/Glycine_max/Glyma1/.

**Table 3:** Soybean gene models and their Arabidopsis annotations within a syntenic region near the 1_0804 locus

Soybean Chromosome	Soybean gene model	Homologous cowpea locus	E-score	Arabidopsis annotation
Gm11	Glyma11g35090.1	-	1.00E-174	CHR2V8|COORD:16735456..16738238| similar to proline-rich family protein [Arabidopsis thaliana] (TAIR:AT3G09000.1)
Gm11	Glyma11g35100.1	-	1.00E-176	CHR5V8|COORD:1712951..1715640| ATE1 (DELAYED LEAF SENESCENCE 1); arginyltransferase
Gm11	Glyma11g35110.2	-	1.00E-59	CHR2V8|COORD:16755237..16756435| yippee family protein
Gm11	Glyma11g35110.1	-	1.00E-59	CHR2V8|COORD:16755237..16756435| yippee family protein
Gm11	Glyma11g35120.1	-	-	-
Gm11	Glyma11g35130.1	1_0804	1.00E-111	CHR2V8|COORD:16752962..16754268| LHCB4.3 (LIGHT HARVESTING COMPLEX PSII); chlorophyll binding
Gm11	Glyma11g35140.1	-	4.00E-24	CHR4V8|COORD:16426775..16427836| TAF8 (TBP-ASSOCIATED FACTOR 8); DNA binding
Gm11	Glyma11g35150.1	-	-	-
Gm11	Glyma11g35160.1	-	-	-
Gm11	Glyma11g35170.1	-	3.00E-67	CHR2V8|COORD:16750185..16751533| similar to unknown protein [Arabidopsis thaliana] (TAIR:AT3G55880.2)
Gm11	Glyma11g35180.1	-	-	-
Gm11	Glyma11g35190.1	-	-	-
Gm11	Glyma11g35200.1	-	-	-
Gm11	Glyma11g35210.1	-	1.00E-149	CHR2V8|COORD:5685088..5687703| ATCHX15 (cation/hydrogen exchanger 15); monovalent cation:proton antiporter
Gm11	Glyma11g35220.1	-	6.00E-11	CHR3V8|COORD:19436813..19438010| SYP122 (syntaxin 122); SNAP receptor
Gm11	Glyma11g35230.1	-	2.00E-11	CHR1V8|COORD:9715602..9720333| RNA helicase, putative
Gm11	Glyma11g35240.1	-	1.00E-113	CHR3V8|COORD:3511107..3512905| GDSL-motif lipase/hydrolase family protein
Gm11	Glyma11g35240.2	-	1.00E-113	CHR3V8|COORD:3511107..3512905| GDSL-motif lipase/hydrolase family protein
Gm11	Glyma11g35250.1	-	1.00E-10	CHR1V8|COORD:6781665..6782015| LCR78/PDF1.4 (Low-molecular-weight cysteine-rich 78)
Gm11	Glyma11g35260.2	-	7.00E-71	CHR1V8|COORD:6585078..6586299| TTN10 (TITAN 10)
Gm11	Glyma11g35260.1	-	7.00E-71	CHR1V8|COORD:6585078..6586299| TTN10 (TITAN 10)
Gm11	Glyma11g35270.1	-	7.00E-28	CHR2V8|COORD:16741623..16741958| ELF4 (EARLY FLOWERING 4)

QTL *Mac-7 *was significant in all three field experiments. In the two experiments conducted with 57 RILs, the QTL peak co-located with SNP marker 1_0678 derived from a homolog of the UDP-glucosyltransferase gene (Table 2). Synteny with *M. truncatula *was identified with a region of chromosome 7 where three copies of pectin esterase-related genes were identified http://www.medicago.org. In the other experiment conducted with 108 RILs, the QTL peak mapped 5.8 cM away and co-located with SNP 1_0153 derived from a lipase class 3 family protein-encoding gene. Based on BLAST searches using this EST sequence, no homology was found in *M. truncatula*, but significant homology was found on soybean chromosome 3. No classic disease resistance associated genes were found near the lipase class 3 protein encoding gene, which was next to homologs of the Photosystem II reaction center W gene. In addition, contiguous loci carrying three copies of genes homologous to the *FLK *(flowering locus KH domain) were identified. LG7 and LG8 which harbored the two maturity QTL based on the AFLP-only map [24] were consolidated into LG5 of the current RIL map and LG4 of the consensus genetic map as described by Muchero et al [25]. EMBL accession numbers and annotation details of cowpea ESTs harboring QTL-associated SNP markers are given in Table 4.

**Table 4:** Annotation of cowpea ESTs harboring SNP markers associated with Macrophomina resistance QTL

SNP marker	Cowpea EST accession number	Best Annotated EMBL hit	E score	Annotation
1_0709	FF388857	DQ446394	3.00E-42	*Arabidopsis thaliana *MATE efflux family protein
1_0551	FG940166	BE554813	4.90E-92	*Glycine max *Endo-xyloglucan transferase
1_0853	FG876090	EE127646	6.84E-74	*Glycine max *Pectinesterase inhibitor
1_0604	FG841619	BE190146	2.90E-72	*Glycine max *Pectinacetylesterase precursor
1_0464	FF544070	CA905170	6.60E-115	*Phaseolus coccineus *Ribosomal protein L7Ae
1_0201	FG818679	AB210817	1.50E-64	*Arabidopsis thaliana *Cell growth defect factor 1
1_0079	FG880038	AF145386	3.90E-84	*Phaseolus vulgaris *CA2+- binding protein 1
1_0496	FG909180	AB027154	3.10E-96	*Vigna unguiculata *Pathogenesis related protein 3
1_0699	FF382329	BG238363	3.30E-114	*Glycine max *Tropinone reductase homolog
1_0804	FC458836	BQ296449	1.90E-96	*Glycine max *Putative Chlorophyll binding protein
1_0678	FG823412	AB070752	1.00E-132	*Vigna angularis *Glycosyltransferase
1_0153	FC457603	FJ461591	9.20E-29	*Brassica napus *Chloroplast Lipase
1_0030	FG935301	AJ133146	1.60E-32	*Persea Americana *Fructose-bisphosphate aldolase
1_0032	FG886549	CA907484	5.80E-59	*Phaseolus coccineus *Protein transport protein SEC61
1_1533	FF549040	XP002881680	2.00e-95	*Arabidopsis thaliana *Integral membrane Yip1 family protein

### QTL pyramiding

**Figure 5:**
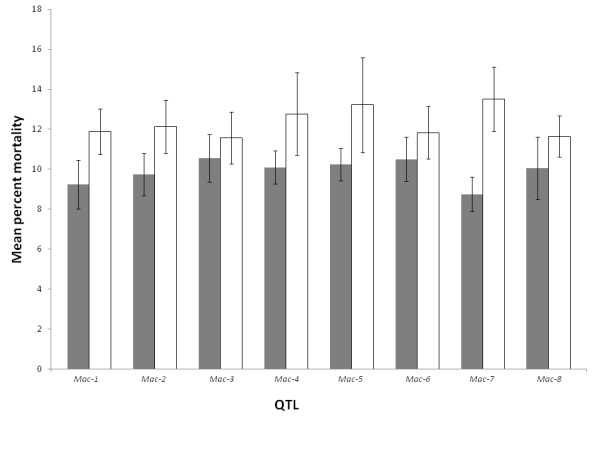
**Single QTL effect on mean percent mortality (± standard error) based on genotypic classes carrying the favorable allele (+, grey bars) or the null genotype (-, white bars) for the field experiment in 2008**.

**Figure 6:**
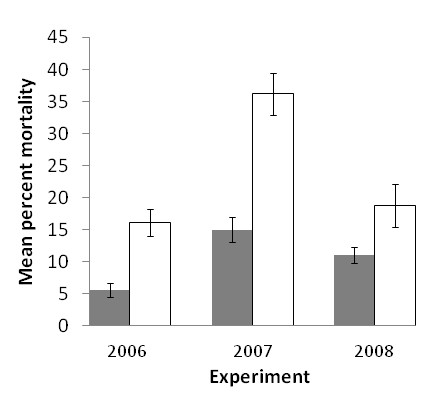
**Effect of the combination of *Mac-2 *and *Mac-5 *QTL on mean percent plant mortality (± standard error) based on genotypic classes carrying both favorable alleles (++, grey bars) and null genotypes (--, white bars) for field experiments in 2006, 2007, and 2008**.

Eight QTLs (*Mac-1 *to *Mac-8*) were selected for the single and pair-wise analysis based on their consistency in map location across experiments. In general, mean percent mortalities for the null genotypes were higher than genotypes with favorable alleles. However, in the analysis based on single QTLs, only *Mac-1*, *Mac-2 *and *Mac-7 *showed statistically significant differences out of the eight QTLs (Figure 5), and 15 out of the 28 pair-wise combinations showed significant differences (Table 5). Percent reductions for the pair-wise combinations ranged from -5.8 to 47.6% with six combinations resulting in a reduction of mean percent mortality greater than 40% (Table 5). Five of the six combinations had QTL *Mac-7 *in common and these ranged from 42.8% to 47.6% reduction in mean percent mortality. The remaining *Mac-2*/*Mac-5 *combination resulted in a 41.0% percent reduction, and the null genotypes for this combination also had the highest mean percent mortality (18.8% ± 3.37%). Individually, *Mac-2 *and *Mac-5 *resulted in reductions of mean percent mortality of 19.6% and 22.5%, respectively, suggesting an additive effect in genotypes carrying both favorable alleles. This QTL combination was chosen for further analysis using the 2006 and 2007 data. The *Mac-2*/*Mac-5 *combination resulted in a 65.6% reduction in mean percent mortality based on 2006 data and 58.5% for the 2007 data (Figure 6). Although they exhibited the largest reductions on percent mortality, QTL combinations with *Mac-7 *were not favored for this analysis due to potential linkage or pleiotropic effects of the *Mac-7 *locus with maturity which may limit its practical use.

**Table 5:** Pair-wise analysis of the effect of QTL combinations on mean percent mortality between genotypic classes

QTL combination	-- genotype			++ genotype			% reduction in mortality
			
	Mean % mortality	N	S.E	Mean % mortality	N	S.E	
*Mac-1/Mac-7*	14.3	30	2.07	7.5	17	1.38	47.6
*Mac-5/Mac-7*	15.8	15	3.5	8.4	42	0.83	46.8
*Mac-2/Mac-7*	13.7	25	2.35	7.7	28	1.06	43.8
*Mac-4/Mac-7*	15.1	19	2.97	8.5	41	0.85	43.7
*Mac-3/Mac-7*	14.5	24	2.34	8.3	24	1.38	42.8
*Mac-2/Mac-5*	18.8	15	3.37	11.1	34	1.25	41.0
*Mac-2/Mac-4*	17.8	16	3.45	10.9	29	1.4	38.8
*Mac-5/Mac-6*	16.4	13	4.06	10.2	28	1.23	37.8
*Mac-1/Mac-4*	15.6	20	2.91	10	18	1.56	35.9
*Mac-1/Mac-5*	16.3	19	2.94	10.5	23	1.41	35.6
*Mac-1/Mac-2*	12.8	39	1.69	8.6	17	1.78	32.8
*Mac-7/Mac-8*	13.4	15	3.3	9.4	33	1.23	29.9 ns
*Mac-3/Mac-5*	14.5	16	3.2	10.2	35	1.17	29.7 ns
*Mac-2/Mac-6*	11.9	34	1.84	8.38	23	1.15	29.6
*Mac-4/Mac-6*	14.7	19	3.05	10.4	28	1.24	29.3
*Mac-4/Mac-5*	13.9	21	2.91	10	60	0.88	28.1
*Mac-4/Mac-8*	15.7	9	5.07	11.4	39	1.12	27.4 ns
*Mac-6/Mac-7*	13.9	34	1.9	10.2	33	1.22	26.6
*Mac-3/Mac-4*	13.1	25	2.25	10	37	1.07	23.7 ns
*Mac-5/Mac-8*	14.9	10	4.54	11.4	46	1.08	23.5 ns
*Mac-1/Mac-6*	13	39	1.69	10	11	2.01	23.1 ns
*Mac-1/Mac-3*	13.2	34	1.8	10.3	12	2.24	22 ns
*Mac-2/Mac-3*	12.3	29	1.9	9.8	23	1.51	20.3 ns
*Mac-3/Mac-6*	12.4	32	1.83	10.8	20	1.47	12.9 ns
*Mac-1/Mac-8*	11.2	22	2.3	10.1	17	1.61	9.8 ns
*Mac-2/Mac-8*	12.3	25	2.06	11.3	34	1.26	7.8 ns
*Mac-6/Mac-8*	10.4	24	2.18	11	28	1.39	-5.8 ns
*Mac-3/Mac-8*	10.6	18	2.31	11.3	28	1.35	-6.6 ns

(--) lack both favorable alleles; (++) carry both favorable alleles; N = number of RILs in each genotypic class; S.E. = standard error;

ns = not significant at the 0.05 probability level.

### Co-location of drought and *Macrophomina *resistance QTLs

Co-location was observed between *Mac-4*, *Mac-5*, and *Mac-9 *with seedling drought response QTLs *Dro-5*, *Dro-10*, and *Dro-7*, respectively, which were mapped previously [24]. In each case, the *Macrophomina *resistance haplotype in these loci corresponded with the haplotype for tolerance to seedling-stage drought stress as measured by delayed drought-induced premature senescence. Soybean genomic regions syntenic to the 3 QTL regions harbored osmotic stress responsive genes such as heat shock, calcium sensing and sodium hypersensitive genes. However, the region syntenic to *Mac-5 *QTL region also harbored disease resistance/susceptibility genes such as glycoside hydrolase, lipase, pectinase, and pectin esterase genes.

## Discussion

Important sources of genetic resistance to *Macrophomina *were identified in cowpea genotypes assessed under mild drought-stress conditions. Based on field screening, genotypes IT98K-499-39, Suvita 2, IT93K-503-1, and Mouride exhibited the highest levels of resistance and are potential sources of host resistance in management strategies in response to *Macrophomina *infection.

Screening a RIL population derived from a cross between the resistant IT93K-503-1 and susceptible CB46 genotypes facilitated the genetic analysis of the resistance trait in one of these highly resistant germplasm sources. Our QTL mapping approach took advantage of the recently developed high density EST-derived SNP marker consensus map of cowpea [25] in which the IT93K-503-1 × CB46 RIL was a constituent map. The QTL mapping indicated a quantitative basis of this trait and also transgressive segregation for both resistance and susceptibility. The transgressive lines with higher resistance levels indicate the potential for selecting novel resistance forms in breeding populations in which the susceptible parent contributes to the resistance phenotype.

Furthermore, genic SNP markers with disease resistance gene annotation and disease resistance-associated genes were found in the genomic intervals defined by the QTL and these were located within syntenic regions in soybean and *Medicago*. Most interestingly, pectin metabolism related genes were identified in three major QTL intervals. The role of plant cell wall polysaccharides, including pectins, in defense against pathogenic microbes has been described [26], and Radha [27] demonstrated that French bean plants infected with *Macrophomina *had 50% less pectin compared to healthy ones, an observation attributed to the pectinolytic activity of enzymes released by the pathogen. Examples of the role of pectins in pathogen defense in other systems include the over expression of pectin methylesterase inhibitors in *Arabidopsis *that resulted in increased resistance to the necrotrophic fungus *Botrytis cinerea *[28]. Similarly, disrupting a pectin methylesterase gene resulted in reduced virulence of *B. cinerea *on apple (*Malus domestica*), grapevine (*Vitis vinifera*) and *Arabidopsis *[29]. Since *Macrophomina *and *B. cinerea *share similar pathogenicity mechanisms involving pectin degradation as described above, these results suggest an important role for pectin-related genes in defense against *Macrophomina*. However, this cannot be addressed in the present study, and additional functional genetic studies will be required to validate this potential role. QTL *Mac-2 *coinciding with a pectin esterase inhibitor had the highest level of expression and statistical significance of all eleven resistance loci mapped in this study, making it a suitable target for molecular characterization. The significant effect of *Mac-2 *on *Macrophomina *resistance will likely result in noticeable and quantifiable change in phenotype sufficient to support its role as a Mendelian factor. Several other QTL mapped in intervals where disease resistance genes were found based on the cowpea genic-SNP markers and synteny with soybean and *Medicago*. These provide excellent additional candidate gene targets for functional characterization.

This study demonstrated the co-location of *Macrophomina *resistance and maturity related senescence QTL in the RIL population used for mapping. This finding in cowpea supports the suggested association between early maturity and susceptibility to *Macrophomina *in other crops [22,19,23]. The same pattern of association between earliness and susceptibility to fungal pathogens has been described in other systems as well. Notably, early maturing potato cultivars have been shown to be more susceptible to the late blight disease caused by the fungal pathogen *Phytophthora infestans *[30-32]. Similar to our findings, QTLs independent of maturity effects have been reported providing the opportunity to breed for early-maturing, late blight-resistant potato cultivars [31,32]. Gebhardt and Valkonen [33] suggested that this association was pleiotropic in nature with the same genes affecting both traits in potato. Interestingly, based on cowpea synteny with soybean and *Medicago*, both flowering and chlorophyll-metabolism related candidate genes were identified within the two maturity-associated QTL intervals and no apparent disease resistance genes were identified. Although this does not exclude the role of other genes within these QTL intervals, these findings may provide additional molecular evidence for the association between early maturity and *Macrophomina *susceptibility that has been described previously.

The association between early maturity and *Macrophomina *susceptibility was demonstrated further by the locus carrying *Mac-7 *and *Mat-1*. This locus had the largest effect on maturity-induced senescence in a previous study [24], and in the current study, also had the strongest effect on mean percent mortality in both single locus and pair-wise loci analyses. However, the co-location of the maturity and *Macrophomina *response QTL on this locus may limit its potential application in marker-assisted breeding programs due to tight linkage and possible pleiotropic effects. Breeding for *Macrophomina *resistant early-maturing varieties that are an important drought escape strategy in parts of West Africa will be complicated by the opposite effects that this locus confers to earliness and *Macrophomina *resistance. In our simulation study we selected *Mac-2 *and *Mac-5 *QTLs as possible target QTLs for pyramiding to improve *Macrophomina *resistance. The presence of these two QTLs resulted in a doubling in reduction of *Macrophomina*-induced mean percent mortality compared to the presence of either QTL individually. This gain in effect was, however, much less significant (gain of 1% - 3%) when we added a third QTL to this combination, suggesting that for breeding purposes, the value of pyramiding three loci compared to two may be markedly diminished.

Genetic overlap was observed in cowpea only between three QTLs mapped in response to drought-induced premature senescence and response to *Macrophomina *infection. This represented a relatively small proportion of overlap considering the ten QTLs mapped for the drought response [24] and nine QTLs mapped here for response to *Macrophomina *infection. Whether this co-location is due to linkage or pleiotropy will require further experimentation especially for *Mac-5 *QTL where both osmotic stress and disease resistance genes were found in the corresponding syntenic soybean genomic region. Similar findings were reported in sorghum where the non-senescence drought tolerance trait was shown to be largely independent from the *Macrophomina *resistance trait [13]. These findings have important implications for cowpea breeding programs aimed at improving productivity under arid and semi-arid conditions. *Macrophomina *can cause significant crop losses even under mild drought stress conditions, and introgression of *Macrophomina *resistance loci should constitute an important component of the breeding schemes targeting genetic improvement with drought tolerance. Furthermore, because early maturing varieties are used as a drought escape strategy in rain-fed production systems, the introgression of non-maturity related resistance loci will be required to mitigate their susceptibility.

## Conclusion

Important sources of *Macrophomina phaseolina *resistance in cowpea were identified and QTLs for trait determinants of the resistance were located on the EST-derived SNP map. Our observations of candidate genes in the QTL regions with annotations commensurate with the respective phenotype used in revealing the QTL, support this QTL-based approach in identifying genetic determinants of important traits in cowpea. The added resolution provided by synteny with reference legumes makes this approach highly effective. However, because numerous candidates were identified for each QTL, there is a need for effective complementary strategies such as virus-induced gene silencing (VIGS) to validate gene function. Functional analysis also will require a robust greenhouse screening protocol, which based on the inconsistent correlation with field experiments, must be optimized to facilitate such studies. The quantitative nature of *Macrophomina *resistance highlighted by the number of QTL identified in this study as well as the relatively low contribution of individual loci toward overall resistance makes breeding for this trait difficult. As such, targeting subsets of loci with higher cumulative effects followed by marker-assisted breeding offers an avenue to start incorporating resistance in economically important cultivars. However, the advent of high density marker-based breeding approaches such as whole-genome and genome-wide selection should enhance the ability of breeders to introgress numerous QTL within the context of the same breeding scheme.

## Methods

### Plant material

Fourteen diverse cowpea genotypes were evaluated in two field experiments conducted in 2006 and 2007. The following genotypes were chosen from the University of California, Riverside cowpea germplasm collection: IT98K-499-39, Suvita 2, Mouride, Bambey 21, UCR24, IT98K-556-6, UCR232 (IT82E-18), CB27, CB46, IT93K-503-1, UCR779, 24-125B-1, 524B, and IT84S-2049. Lines having an 'IT' prefix are advanced breeding lines developed at the International Institute of Tropical Agriculture (IITA) whereas lines having a 'CB' prefix and UCR24 are cultivars and breeding lines developed in California for production of blackeye-type cowpeas. Genotype 24-125B-1 is an advanced breeding line developed by Institute of Agricultural Research for Development (IRAD) in Cameroon and UCR779 is a landrace accession from Botswana. The accessions represented genotypes with prostrate and erect growth habits, early and late-maturity, and photoperiod sensitivity and insensitivity. These genotypes also belong to high, moderate and low seedling drought tolerance classes as described by Muchero et al [34]. In addition, 108-F_2:8 _RILs developed by single seed descent from a cross between the drought tolerant medium-maturing breeding line IT93K-503-1 and drought susceptible early-maturing CB46 genotypes were evaluated in three field and two greenhouse experiments conducted in 2006, 2007, and 2008 for QTL mapping.

### Field experiments

Three field experiments were conducted from June to September of 2006, 2007, and 2008 at the Citrus Research Center-Agricultural Experiment Station (CRC-AES) of the University of California Riverside (33°57'54''N; 117°20'08''W), USA. The experiments were planted alternately in 2 adjacent field plots with a history of *Macrophomina *infestation and long-term cultivation of cowpea crops. The soil is classified as an Arlington fine sandy loam (coarse-loamy, mixed, thermic Haplic Durixeralf) [35]. The quick surface-sealing properties of this soil type made it suitable for simulating a gradual and mild drought stress conducive to *Macrophomina *infection without the confounding effects of overly rapid onset of severe drought stress.

For all field experiments, 200 greenhouse-grown seeds per genotype were distributed equally in 5 replicates, each planted on 5-m-long plots spaced 0.75 m apart. A subset of 57 RILs, two parental genotypes, and 14 diverse genotypes were evaluated in 2006 and 2007, whereas 108 RILs, which included all the 57 RILs evaluated before, and parental genotypes were evaluated in 2008. The experimental design was a randomized complete block. Drought stress was imposed by growing the crop with only two irrigations, a pre-irrigation about 7 days prior to planting, and a single post-planting irrigation applied 7 days after emergence. These irrigations were applied to furrows and of sufficient duration to fill the soil profile. No further irrigations were applied to the stress treatment. In 2006 and 2007, adjacent blocks having the same experimental material were grown under 'full' irrigation by applying irrigation to field capacity once every seven days for the duration of the active growing period. The well-watered block had 4 replicates and was considered as the *Macrophomina *plus non-stress treatment, while the stressed field block was considered the *Macrophomina *plus drought-stress treatment. The same set-up was adopted in 2008 except only RILs and the parental genotypes were evaluated and the well-watered comparison block was not included. Seedling emergence for the *Macrophomina *plus drought-stress treatment was recorded one week after planting. *Macrophomina *infection was allowed to occur naturally after which disease evaluation was conducted after 3 to 4 weeks from planting when symptoms of infection started to occur. At this stage, plant mortality counts were recorded for each plot every 7 days until the budding stage. Percent mortality was calculated using the final cumulative mortality counts for each RIL and genotype using the formula: % mortality = (total mortality/total emergence) ×100.

### Greenhouse experiments

#### (a) *Macrophomina *isolation and inoculum preparation

Isolation and identification of *M. phaseolina *was carried out using the method described by Aboshosha et al [36] with minor modifications. Briefly, diseased cowpea stems displaying typical ashy stem blight symptoms were collected from the infested CRC-AES field. One-cm stem sections were cut, soaked in concentrated bleach solution for 3 min, and then washed thoroughly under running de-ionized water. Cut sections were placed on sterile potato dextrose agar (PDA) plates and incubated for 3 days at 35°C. Colony identification was primarily based on morphology and visualization of the characteristic black irregularly shaped microsclerotia. 5-mm-diameter disks were cut from each PDA plate, transferred to a new PDA plate and incubated at 35°C for three days. The same procedure was repeated twice. Finally, ten 1-cm^2 ^PDA plugs with microsclerotia were kept in sterile deionized water at room temperature for long-term storage. Koch's postulates were fulfilled using the isolated *M. phaseolina *cultures by inoculating greenhouse grown cowpea plants, observing disease development and re-isolating the fungal pathogen from diseased plants.

Inoculum for greenhouse studies was prepared by transferring a 1-cm^2 ^plug from long-term storage to a fresh PDA plate. PDA plates were incubated for 5 days at 35°C for maximum microsclerotia development. PDA with fungal mycelia and microsclerotia was then macerated in sterile water and filtered using quadruple layer cheesecloth to collect the microsclerotial suspension. Microsclerotia were counted under a light microscope and the suspension was adjusted to a final predetermined inoculum concentration of 250 ± 10 microsclerotia per ml de-ionized water.

#### (b) Resistance screening

Genotypes IT93K-503-1, CB46 and the set of 57 RILs were used in the greenhouse experiments. Greenhouse temperatures were maintained at 30°C during the day and 25°C during the night. Each experiment was carried out using 878-ml plastic pots filled with 750 g of steam-sterilized UC-MIX B [37] potting media. Each experiment consisted of 4 replicates in a randomized complete block design. Pots were watered to capacity and then planted with 2 greenhouse-grown seeds. Five days after planting, pots were thinned to 1 plant per pot and watered to capacity for the last time. Seven days after the last watering when drought stress was becoming noticeable through leaf curling of unifoliates, two-week old plants were inoculated with a microsclerotia suspension at 250 ± 10 microsclerotia/ml. Two 5-mm parallel cuts were made 1 cm below the soil line on each plant stem. 1 ml inoculum suspension was pipetted directly onto the exposed cuts and immediately covered with potting media. Disease development was evaluated 7 days after inoculation when *Macrophomina*-induced wilting and stem discoloration started to appear. From this stage, plants were rated every 3 days for the characteristic ashy-stem symptom by rating the progression from the soil line up the stem of stem discoloration resulting from infection. Symptoms appeared as a deep red to brown lesion that was visible initially just above the soil line. Disease severity was scored on a scale of 1 to 10 (0-1 = no lesion to slight discoloration at soil line; 1-2 = lesion above soil line but confined below midpoint of first node; 2-3 = lesion extends up to first internode; 3-4 = lesion extends beyond first internode but confined to second node; 4-5 = lesion more than half way up second node; 5-6 = lesion extends beyond second internode but confined to third node; 6-7 = lesion extends over third internode; 7-10 lesion extends up to the growing point).

### QTL Analysis

QTL analysis was conducted with the Kruskal-Wallis and Multiple-QTL Model Mapping (MQM) packages of MapQTL 4.0 [38] using a combined AFLP [24] and SNP marker-based [25] genetic linkage map of the IT93K-503-1 × CB46 RIL population. Percent mortality data of the RIL population were used to identify QTLs associated with response to infection under field conditions and lesion severity ratings of the RILs were used to identify loci for response to infection under greenhouse conditions. QTLs were considered significant if the same QTL interval was detected in more than one experiment with at least two experiments meeting the Kruskal-Wallis significance threshold or at least one experiment meeting the calculated LOD significance threshold as described below. Significance thresholds were set at the more stringent 0.005 for Kruskal-Wallis as suggested by the authors of the software [38]. Logarithm of odds (LOD) thresholds for MQM analysis were calculated for each experiment and each linkage group using 1000 permutations at the 0.05 significance level. Graphical representation of QTL was done using Map Chart 2.2 software [39].

Placement of *Macrophomina *resistance QTL on the cowpea high-density consensus map [25] was facilitated by common SNP markers between the IT93K-503-1 × CB46 map and the consensus map. Candidate gene identification was facilitated by the cowpea consensus map-genic SNPs from the HarvEST:Cowpea browser (http://www.harvest-web.org) as well as *Glycine max *and *Medicago truncatula *synteny relationships described by Muchero et al [25]. Cowpea candidate gene ESTs were annotated based on best hit BLAST searches [40] in the EMBL nucleic acid sequence database.

### Statistical Analysis

Data were analyzed for frequency distribution and Spearman Rank correlation using the Statistix 8.0 software [41]. The effect of single and pair-wise QTL combinations on mean percent mortality was done by classifying RILs into genotypic classes with favorable alleles (+ or ++) and null genotype (- or --). Class-specific means of percent mortality and standard errors were calculated for each genotypic class. This analysis was conducted using cumulative percent mortality from the 2008 experiment. Heterozygosity at the locus in question was considered as missing data and was excluded from the analysis. Single or double QTL effectiveness was evaluated based on the degree of reduction in percent mortality of the null compared to genotypes with favorable alleles. QTL combinations that resulted in the largest mortality reductions were examined further using data from the 2006 and 2007 field experiments in which 57 RILs were sub-divided into genotypic classes as described above. SNP markers 1_0709 (*Mac-1*), 1_0853 (*Mac-2*), 1_0604 (*Mac-3*), 1_0201 (*Mac-4*), 1_0079 (*Mac-5*), 1_0804 (*Mac-6*), 1_0678 (*Mac-7*), and 1_0030 (*Mac-8*) were used in the genotypic classification exercise based on their association with the respective QTL (Table 2).

## Authors' contributions

WM, JDE, and PAR conceived and designed the experiment. WM and JDE conducted the experiment. WM, JDE, TJC, and PAR analyzed the data. WM, JDE, TJC, and PAR wrote the paper. All authors read and approved the final manuscript.
